# “Bringing new life in”: Hope as a know-how of not knowing

**DOI:** 10.3389/fpsyg.2022.948317

**Published:** 2022-12-14

**Authors:** Elena Cuffari, George Fourlas, Maceo Whatley

**Affiliations:** ^1^Scientific and Philosophical Studies of Mind Program, Department of Psychology, Franklin and Marshall College, Lancaster, PA, United States; ^2^Hampshire College, Amherst, MA, United States

**Keywords:** hope, parenting, know-how, crisis, reflective lifeworld research, languaging, enactivism, ecological psychology

## Abstract

We offer a theoretical and empirical exploration of parental or guardian hope through an enactive, ecological, and reflective lifeworld research framework. We examine hoping as a practice, or know-how, by exploring the shape of interviewees’ lives as they prepare for lives to come. We pursue hoping as a necessarily shared practice–a social agency–rather than an individual emotion. One main argument is that hoping operates as a kind of languaging. An enactive-ecological approach shifts scholarly conversations around hope, in part by including voices of non-scholars and considering lifeworld factors like class privilege. We aim to identify particular impediments to or facilitators of hope, which may be thought of as classes of restrictive and generative thought-shapers, respectively. Results from our qualitative study indicate that uncertainty is deeply salient to hoping, not only because hope as a concept entails epistemic limits, but more vitally because not knowing, when done skillfully and when supported through education and some degree of socio-economic security, leaves room for others to reframe utterances, and so for the family or community to resist linguistic enclosure.

## Introduction

In the present study we consider the question of enacting hope, and more specifically, how parents or guardians hope. We look for illumination of hope as a practice by exploring the shape of our interviewees’ lives as they prepare for lives to come--their own as well as the lives of their soon to be or recently born, much hoped for, or distant children. Following from enactive premises of participatory sense-making and linguistic bodies theory, we pursue hope as a necessarily shared practice, that is, a social agency, rather than solely as an individual emotion. We explore hoping as a kind of languaging, with all of the attendant powers, sensitivities, and entanglements involved in this self, other, and, world relating behavior.^1^ Following from ecological psychological premises, we also explore hope as an objective feature of a situation. “Our habits of mind–broadly speaking, our characteristic ways of attending to, interpreting, and engaging with the world–are ecologically structured” (Krueger and Maiese, 2018, 13); languaging, on our account, is a habitual practice of environmental engagement. These two theoretical starting points jointly steer us away from the most traditional approaches to hope in philosophy and moral psychology. Taken together, an enactive-ecological approach to hoping as a situated social practice may aid in identifying particular impediments to or facilitators of hope, which may be thought of as classes of restrictive and generative thought-shapers, respectively.

This article proceeds in two broad stages. We first offer a theoretical discussion of hope (Sections “Introduction” and “Sketching an enactive and ecologically informed approach to hope practices”), on which we base our broad hypothesis that the know-how of hoping consists in resisting linguistic enclosure, the rigid closing-off of possibilities that happens in overly deterministic naming, labeling, pronouncing, knowing-that. We also hypothesize that hope is a salient feature of pregnancy (as well as parenting) as it is a time marked by expectation and uncertainty on many timescales (the pregnancy itself, labor and birth, postpartum, caring for a newborn, raising a child).^2^ We take the experiences of new parents/guardians as a paradigm case of what one of our study participants describes as “bringing new life in,” that is, participating actively in not-knowing, which requires and makes space for confrontation with the virtual self (Varela, 1999). We then present an original, exploratory investigation of hope practices (Section “Parent-guardian hope practices: A qualitative study”) along with findings that expecting parents and guardians from a certain lifeworld experience hopefulness as a function of realizing unknowing. These participants enact hope in jointly, linguistically reframing situations toward greater possibility. We discuss hoping as it relates to privilege (Section “Discussion”) and conclude (Section “Conclusion”) by reflecting back on our initial theoretical posits.

### Hope: How and why, not what

We want to take a step back from the deluge of attempts to define and dissect hope, to offer prophetic pronouncements about it, and to specify exhaustively and in advance the ways it can and cannot take shape. And yet, to talk about what blocks something or what facilitates it, one does need some operative notion of what the thing is, so that it can be identified. Perhaps with a little bit of “tuning” (Morton, 2018) most readers can think of stories, songs, characters, and most of all their own experiences, and know what hope “really” is. Some of this might seem initially counterintuitive, but let us at least try to explain our path and see if and where we and the reader may meet.

Hope is there when Tig, a young millennial biologist too keenly aware of what it means to “throw shit out,” pins a cloth diaper onto her nephew in Unsheltered (Kingsolver, 2018). It is there when Nick in the Great Gatsby narrates and co-experiences the implosion of a small high-society community in 1920s United States: when in conversation with others or when in a position to observe their most flagrant missteps, he heeds his father’s advice that “reserving judgment is a matter of infinite hope” (Fitzgerald, 1925). It is there in the astonishing mettle of Essun/Damaya/Syenite, the 40-year-old mother, orogene, and climate refugee guiding one found family after another through the falling ash of the longest apocalypse in The Broken Earth trilogy (Jemisin, 2015, 2016, 2017). These characters embody the qualities we identify as hopeful.^3^

Hopeful qualities could be described in various ways. Following theoretical and empirical work we provide below, we suggest that what being hopeful boils down to is enacting a paradox: one simultaneously lets go of the illusion of total agency and complete knowledge, while remaining a full participant in the unfolding situation. Note that stories allow room for the embodying and unfolding of a hope practice, which is idiosyncratic and nuanced, and cannot be reduced to a single proposition. Single sentence definitions run into trouble because so many of them sound good but stand in contradiction to each other.

Additionally, we suggest not thinking about hope (only) as an emotion.^4^ We can feel full of hope of full of despair, or many things in between, to be sure. Being an adult, a parent, a person, is a perpetually difficult business. Little in our society or world right now is making that easier, if one can even meaningfully quantify or compare the hopelessness entailed in the global climate crisis, the global pandemic, the global struggle with authoritarianism, racism, misogyny, and capitalism. But this is our point: hope is a part of shared reality, to a greater or lesser degree. Tied up with this, and perhaps part of why we find people often interrupting each other’s expression of despair (whether through advice or comfort giving, or more formally as therapists, doctors, teachers, or activists), is that our acts respond to and shape reality. What we say, think, and do, can be toward hope, emerge out of hope, or generate hope (or not). Hope is a stance or a way of being, one which can be expressed (or not) in languaging, thinking, interacting, and working. This hope stance is what we want to consider.

### Tensions in hope scholarship

Hope is a much-treated topic in philosophical and psychological research in the past three decades (Van den Heuvel, 2020) and increasingly in monographs, long-form think pieces, or editorials for public consumption over the past several years (West, 2008; Gay, 2019; Solnit, 2020), as the state of world affairs grows increasingly desperate, especially for privileged populations (white, Western, wealthy, etc.) that were previously sheltered from climate disasters, economic peril, and other forms of hardship. The prevailing formula for defining hope as “desire + uncertainty” (Blöser and Stahl, 2019; Van den Heuvel, 2020) is not satisfying enough to stave off continued academic disagreement (see Pettit (2004), Martin (2013)), while public pronouncements on hope are contradictory or quickly taken out of context, adage-like. Adrienne Martin rejects the “orthodox” definition in favor of an account that holds that “hoping for an outcome involves standing ready to offer a certain kind of justificatory rationale for engaging in certain kinds of thought, feeling, and planning” (2013, 11). In a sophisticated extension of Kant, Martin makes a case for hope being epistemically and not only pragmatically rational (ibid). However, we find intersubjectivity, the precondition of any claims to or acts of rationality, to depend not only on explicit, self-aware exchanges of reasons but importantly on intercorporeality and shared bodily being. Mainstream moral psychology only glancingly engages with embodied cognitive science approaches, for example, Martin’s theorizing of interpersonal hope draws on extended cognition to argue that “when we invest hope in each other… what we hope for is to extend our agency through each other” (Martin, 2019). This kind of “social” move remains individual, leaving untreated cases of hoping together, hoping as a collaborative act of becoming.

We note four unresolved tensions in the literature: (1) whether or not real hope has an object, (2) whether hoping is a virtuous activity or basic cognitive activity, (3) whether or not hope is contingent upon external circumstance (as opposed to simply being a matter of will), and (4) whether hope is politically desirable.

In philosophy, one finds a view, for example in the work of Gabriel Marcel, that outcome-directed hope (hoping that such and such comes to be) is a deficient or non-essential form of hoping (Michener, 2020). True hope is necessarily open-ended, not self-interested striving but a manner of being in the world: “I hope.” The thinking here, for Marcel, is that attachment to a particular end runs the risk of closing off a present, which may be full of possibilities. In attachment to the end we only experience fear of losing it. In a recent talk, Evan Thompson follows Marcel on this point, saying, “In other words, part of the inner creative process in hope–part of the practice of enacting unconditional hope–is to not allow yourself to get sucked into imagining–I would prefer to say fantasizing–a hoped-for object. For hope to be unconditional, it must transcend fantasy becoming a hope without craving” (Thompson, 2021).

While some contemporary philosophers hold an outcome, in its degree of uncertainty and its degree of desirability, as the key variable of a hope formula, others turn to ancient Western thought and propose that hope is best seen as a virtue, and perhaps a foundational one (Gravlee, 2000; Woolfrey, 2016). Being hopeful (“having the virtue of hopefulness”) “gives us the motivation to see that acting appropriately (virtuously) matters” (Woolfrey, 2016, 128). On this view, hopefulness is a cultivated character trait that underlies and promotes other virtues, such as courage, and that enables hope itself: “Hopefulness precedes and produces the commitment to hope in particular instances” (Woolfrey, 2016, 131). Broadened to the point of background condition, however, one finds a counter perspective that hope cannot be a virtue, as it is unremarkable, indeed a prerequisite for any living or acting at all (McGreer, 2004).

We have identified a philosophical perspective that true, virtuous hope is without object. Put differently, in a “proper” hope stance, one hopes, rather than hopes that or hopes for. This view stands in some tension with the dominant view in psychology, that hope–“rainbows in the mind”–is a product of “pathways thinking” (Snyder, 2002). Hopeful people are those who can see paths of action available to themselves. Significantly, hope comes from the belief that one will be able to meet one’s goal. Rather than the open-ended “I hope,” hope arises from a feeling that “I can.” Hope is explicitly goal-oriented or teleological. From a psychological perspective, not having goals is a mark of depression and despair. The goals themselves do not matter, so long as one is capable of pathways thinking. Hope is thus deemed an “equal opportunity emotion” (Lopez, 2013). Here we would mark another tension. While it seems logical to say that any person can be hopeful–surely, there are so many instances of the human spirit triumphing in abysmal conditions–this also runs up against an at least equally plausible idea (with equal ease of examples that may come to mind) that poverty and oppression provoke despair, or block hope.

Consider the phenomenon that economists Case and Deaton have labeled ‘‘deaths of despair’’ (2020). They found that mortality rates for middle-aged white Americans not only stopped falling in the late 1990s, but began to rise.^5^ Digging deeper, this statistical change appears to be driven by suicides, overdoses, and longer-term demise due to alcoholic liver failure. These “deaths of despair” befall a particular subset of the noted population: those without a college degree, effectively barred from middle class life today. As Case and Deaton’s term suggests, however, this means being cut off from something that middle class life provides, perhaps marginally, but significantly enough to mark the dividing line between life and death, between hope and despair. We will have more to say about this research below.

As alluded to briefly above, in popular discourse drifting a bit beyond the ivory tower, one finds contradictory statements about hope. Hope is criticized as antithetical to action in Roxanne Gay’s op-ed “The Case Against Hope”: “Hope allows us to leave what is possible in the hands of others.” (Gay, 2019). American Tibetan Buddhist nun and writer Pema Chödrön cautions against hope for being a rejection rather than a compassionate embrace of the present moment: “Hope and fear come from feeling that we lack something; they come from a sense of poverty. We can’t simply relax with ourselves. We hold on to hope, and hope robs us of the present moment” (1996, 41). Rebecca Solnit also divides hope from action, but, as we will, she links hope to unknowing: “Hope is an embrace of the unknown and the unknowable, an alternative to the certainty of both optimists and pessimists” (Solnit, 2016). Cornel West’s words come close to our view. West, in contrasting hope with optimism, writes “Hope enacts the stance of the participant who actively struggles against the evidence” (West, 2004, 296, as cited in Stitzlein (2019), 97).^6^

In our study below, we observe the practice of hope as a challenge to the status quo of received information and its framing. Enacting hope does not reject the present, but a foreclosing, overly determining view of the present. This leads us to posit several formulations of what hoping is as a social practice. For example, we will say it is a stance that shapes sense-making, a way of being in the present (a stance-taking in the present), a way of escaping overly determined linguistic subjectivity, a local project of finding more-ness in the present, and a paradoxical practice of staying in participation while letting go. These are our working definitions guiding what follows. We return to evaluate them in our conclusion.

One of our main arguments will be that hope is a linguistic practice. It is important, therefore, for us to specify what we mean by “linguistic.” Ours is a specific use of the term that points to linguistic bodies theory (Cuffari et al., 2015; Di Paolo et al., 2018). We describe this view in the following section. Here we note that our use of ‘‘linguistic’’ does not intend to align us with the extant scientific field that systematically studies languages. We are instead applying the enactive notion of the term, which holds that precarious historical bodies in interaction are the source and product of languaging.^7^ The job of enactive linguistic science, then, is to explain how language–understood as “perpetual managing of an open-ended tension that recapitulates at various levels of participatory sense-making” (Cuffari, 2020,149)–emerges from how these bodies live, what they do, and what they are.

## Sketching an enactive and ecologically informed approach to hope practices

Without claiming to settle any of these issues regarding a final, fixed nature of hope, we suggest a hopefully helpful reframing in the form of an enactive-ecological exploration of hope practices and the conditions of possibility for such practices, which we think steers a middle path between several of the above-noted tensions.

To develop the hope stance we mention earlier, we seek to investigate hoping or enacting hope. We see this as a co-achievement, an enacting of social agency. In other words, hoping is something that linguistic bodies do (Di Paolo et al., 2018). Being enlanguaged, and maneuvering one’s lifeworld through acts of language, is the basic backdrop against which a practice of hope becomes possible, and desirable. The complexity of any person’s lifeworld stands reciprocally co-defines the complexity of that person’s sense-making, their cognition, and their construction of meaning. Hope shows up here as a stance that shapes sense-making, as a manner of moving and being in the present horizon.

Linguistic bodies–bodies whose becoming takes place in enlanguaged environments–are entanglements of organic, sensorimotor, and intersubjective bodily domains. We would here borrow Claudia Rankine’s prose-poetic description of her body as a “cupboard” of past experiences (specifically, linguistic experiences) that give contour to and condition-enable perception of features of the present:

“The world is wrong. You can’t put the past behind you. It’s buried in you; it’s turned your flesh into its own cupboard. Not everything remembered is useful but it all comes from the world to be stored in you. Who did what to whom on which day? Who said that? She said what? What did he just do? Did she really just say that? He said what? What did she do? Did I hear what I think I heard? Did that just come out of my mouth, his mouth, your mouth? Do you remember when you sighed?” (Rankine, 2014, 63).

From Rankine we see that linguistic bodies are also entanglements of temporal domains. It is perhaps more difficult to name the futurity of our bodies than the pasts they carry; we may think at first only of aging, decay, or the forward-pushing forces of habit. But this difficulty serves as a useful reminder of what it means to be a linguistic body, which means a body that continually achieves precarious closure (autonomy) at multiple levels through means of symbolic sensitivities and powers. Able to notice when in the day I am hungry, and what sorts of foods feel good for me, I can plan meals for the coming week. This ability is not so simple, in reality: it points to material resources which point to a history of my financial means and my cooking skills, the state of complicated and currently strained food supply chains, and so much more. At the same time that so much past is implied, my body-to-be, with its need for consistent feeding, and my acts-to-come, set into a typically overscheduled calendar week, are just as virtually present as the past is in my present act of meal planning. Linguistic bodies, as meaning-making and meaning-experiencing beings, live in a horizon of temporality where the future and past are ever present. “One of the most important aspects of human awareness is our ability to extend our experience into the future” (Reed, 1996, 143). If hoping is something afforded to linguistic bodies, it is a way of being in the present, or a stance-taking in the present, that makes both past and future what and how they are.

Equally vital, hoping, as enacting a value, is not an internal state but emerges through an interaction between subject and world (in more ecological parlance, perceiver-agent and environment). The possibility of realizing hope depends to a certain extent on whether or not hope is an objective feature of the situation. A rich resource to consider is found in an ecological values-realizing account offered by Hodges (2007), following up on work by Hodges and Baron (1992). Hodges and Baron (1992) “proposed that values are best defined at the level of ecosystems,” as “the global constraints on an ecosystem that underwrite the system dynamics, which are intentional (i.e., directed) without being teleological” (Hodges, 2022, 9). The radical suggestion here is that values are not individual cognitions, but ontological entities that operate on an agent-environment system. Values do not dictate how they will be realized (they are not teleological) but steer a system normatively to figure out their realization (they are intentional).

Hope as a stance enacted by a group of people toward the future is not about desire first and foremost, nor is it a feeling, but a way of escaping overly determined linguistic subjectivity and knowledge, on individual and community levels. Understood in this way, hoping is perhaps a candidate for the ur-value of agency understood as “an ongoing hunt for better ways of knowing, doing, and being” (Hodges, 2022, 10). Such ‘‘escaping’’ is not under any one person’s control, nor is a community’s will enough on its own.^8^ More recently, in the context of the COVID-19 pandemic, Jonathan Lear published an interpretation of Freud’s personal confrontation with the inherent impossibility of the narrative of knowing and progress that humans drift so deftly toward believing (Lear, 2021). On this account, hope is not willed, but comes to those who struggle through loss and grief, who reckon with the fundamentally unknowable in a way that remains participatory even as it is necessarily without “optimal grip” on what is coming.

### Enacting hope as an ethical practice

Our interpretation of enacting hope as a stance of resisting a hardened linguistic self is inspired by Varela’s discussion of linguistic subjectivity and the emergent closure of the ‘‘I’’ at the end of his Ethical Know-How 1999 lectures.^9^ It is useful to consider the relevant passage, which follows a thorough demonstration of the co-emergence of micro-worlds and micro-identities, and neuroscientific evidence for an emergent yet non-localizable sense of self:

“What we call ‘I’ can be analyzed as arising out of our recursive linguistic abilities and their unique capacity for self-description and narrative. …Our sense of a personal ‘I’ can be construed as an ongoing interpretive narrative of some aspects of the parallel activities in our daily life, whence the constant shifts in forms of attention typical of our microidentities. Whence also the relative fragility of its narrative construction.”

“If this narrative ‘I’ is necessarily constituted through language, then it follows that this personal self is linked to life because language cannot but operate as a social phenomenon. In fact, one could go one step further: the selfless ‘I’ is a bridge between the corporeal body which is common to all beings with nervous systems and the social dynamics in which humans live. My ‘I’ is neither private nor public alone, but partakes of both. And so do the kinds of narratives that go with it, such as values, habits, and preferences.” (Varela, 1999, 61–62).

The “I” is most properly understood as “virtual,” although we do not live this way (ibid, 62). Rather, routine linguistic practices, including coupling to others in conversational and other symbolically mediated interactions, continuously enact a “closure” that sustains the illusion. True ethical practice, according to Varela, is learning how to encounter oneself as virtual (ibid, 63).

To this end, Varela offers brief, provocative remarks on therapy and on “non-response to language.” Consider that “[t]he incorporated flows of utterances that make up a linguistic agent are always the joint result of personal enactments and of patterns that live in the community” (Di Paolo et al., 2018, 193). Therapy works by inviting the client-patient to first externalize her flow of self-directed utterances and then by interrupting this flow, thus transforming the frame of the window through which we view ourselves and our world. Therapist and client-patient form a community somewhat apart from the everyday embeddedness of the client-patient. According to Varela, non-response to language as presented in Eastern wisdom traditions creates a space analogous to that offered by therapeutic analysts (1999, 66). Below we share examples from our study of other, daily ways that hoping is done in interrupting, in un-knowing, and to repeat the words of Cornel West, in “active struggle against the evidence.”

For Varela, then, what is most ethical is staying open. What we suggest here, and find corroborated in the qualitative study discussed below, is that the know-how of hoping likewise consists in staying open, in particular, in resisting linguistic enclosure, the rigid closing-off of possibilities that happens in naming, labeling, pronouncing, knowing that.

### Hope in the experience-environment relation

We note a resonance between the linguistic bodies–Varelian approach and that of ecological psychologist Edward Reed, particularly as read and developed by Hodges. Hope for Reed is a form of good, that is, primary, first hand experience (Hodges, 2022, 8). As such, it is precarious, subject to the same “degradation” that Reed thinks threatens primary experience in our present era, in which alienating work, mass consumerism, and mass media continually foist secondary or indirect experiences upon us and block us from direct, agential world relations and from direct, vulnerable encounters with others (Reed, 1996). Hope can be under threat just because hope is a feature of a shared environment objectively (or it is not); part of an existential situation (or not). “…the most important aspect of our experience, hope, is not a subjective feeling but an objective property of our encounters with the world” (ibid, 153). For Reed hope is objective; its conditions are out there to be found (or they are not; the situation is “hopeless” as we say) and once found, our collaboration with these conditions is accompanied by hope as an aspect of experience. Reed explains, “…the growth of personal experience is necessarily social, as well as individual, and therefore a combination of accrued wisdom and genuine openness is a realistic goal” (ibid).

In the case of “deaths of despair,” hope practices are revealed to be precariously afforded. Materiality matters; what is present, perceivable, and possible for a community matters. Case and Deaton’s central “Deaths of Despair” chapter opens with an excerpt from a 2017 PBS interview of Kentucky residents that the authors take to show the links between suicide, drugs, and alcohol, but which also indicates the community as the level where hope is or is not found. Two women who have both lost their husbands are in conversation. The first interviewee’s husband killed himself because of guilt and depression over his son’s drug use. The other describes the fate that befell her husband and his group of friends: “One died with a heart attack, but drug use and alcohol use played all the way through his life. Another one died of cancer, drank up to the very end. And my husband actually had a G-tube in, a feeding tube in, and poured alcohol down his feeding tube until he died” (quoted in Case and Deaton (2020), 37). Underlining their general argument, Case and Deaton note that in Kentucky “the risk of dying in midlife from suicide, accidental drug overdose, or alcoholic liver disease was a third higher than the national average in 2017. But not all Kentuckians were at equal risk. This risk…had risen markedly, but only for those who did not hold a 4-year college degree” (ibid, 49).

Despair is spreading for those with advanced degrees, too. In a recent New York Times article titled “These Climate Scientists are Fed Up and About to Go on Strike,” profiled scientist Bruce C. Glavovic, a professor at Massey University in New Zealand, was quoted: ““We’ve had 26 Conference of the Parties meetings, for heaven’s sake,” he said, referring to the United Nations global warming summits. More scientific reports, another set of charts. “I mean, seriously, what difference is that going to make?”” (Zhong, 2022). In April 2022, a climate activist known in the climate science community died by self-immolation in protest in front of the USA Supreme Court (Cameron, 2022). Reed wrote that “…hope is part of our experience when we detect information that tells us how to reach a goal” (Reed, 1996, 153). If one is a scientist and does not see that practice as affording possibility, but rather perceives a dead end that the work does not or cannot move beyond, the action that enacts hope may indeed be to switch gears (which perhaps begins with a strike). It is an extreme case where one sees no present space for possibility that leads to the final strike of suicide.

Hope practices arise or fail to arise contingent upon the functioning and interplay of a variety of factors, education, and socio-economic status amongst them. Furthermore, “Meaningful work that allows us to develop our own experience” is “the precondition of” hope (Reed, 1996, 153). Reed acknowledges Snyder’s hope account of pathways and agency, but sees this as requiring, as Dewey and Morris saw, institutional and societal change in schools and in workplaces and in systems of democracy (ibid, 155). Hope, on Reed’s view, is primarily about agency understood not simply as goal-achieving-capacity but as a practice that gives rise to experience (Hodges, 2022, 8). We interpret information-detection in Reed’s sense as an indication that there is more to know; not everything is yet known. Enacting hope is a local project of finding that more-ness in the present. This can evade communities and situations entirely, it appears. Yet the paradox of hope’s ever present potential and ever present precarity remains open because, “…though it is not sufficient… an ideal is still necessary…” to achieve a better world (Fourlas, 2022, 113).

Recent remarks by enactive philosopher of mind Evan Thompson converge with our focus on hoping (1) as a shared practice that emerges from and returns to complex exchanges and entanglements between bodies and environments (2) as a know-how of unknowing and (3) as tied to immediate circumstances. Thompson says, “To speak of ‘enacting hope’ already suggests a general answer to our question about what hope is: hope is an act and a way of being. This way of putting things contrasts with thinking of hope as a feeling or even as a state of mind” (2021). As mentioned above, Thompson closely follows the work of existential phenomenologist Gabriel Marcel to describe the way of being that could be characterized as “enacting hope.” For Marcel and Thompson, being unconditionally hopeful is an experience distinct from that of being optimistic, in that optimism entails expecting and desiring a particular good outcome, a particular good, of which in “unconditional hope” one must be willing to let go. In this way “unconditional hope” can be understood as a practice of not being fixated on a certain object or outcome so that our hope can transcend such attainment and allow us to cultivate with the world presently afforded to us. Such hoping provides a more secure experience of the present moment as not defined by personal cravings.

Thompson argues “that we need to enlarge the ‘us’ of ‘I hope in you for us’ to include the more-than-human-world, by which I mean not just non-human life-forms but also the whole planetary biosphere or ecosphere. I submit that enacting hope as we encounter the climate crisis requires an expanded ecological conception of community and correlative enlarged unconditional hope–a hope in Earth for all of us” (2021). Linking hope with action, Thompson employs Joan Halifax’s idea of “wise hope,” which “is not seeing things unrealistically but rather seeing things as they are, including the truth of suffering-both its existence and our capacity to transform it. It’s when we realized we don’t know what will happen that this kind of hope comes alive; in that spaciousness of uncertainty is the very space in which we need to act” (ibid).

The above theoretical reflections offer very broad hypotheses regarding “hope shapers.” Hope as a set of community practices of utterance management is conditioned by environmental constraints and community-level experiences. What encourages hope is the practice of multiple perspective taking, the making explicit of self-directed utterance flows (sharing, making public) in order to loosen their hold on the self, and of course, a complex set of material and historical circumstances that permit agency and perpetual becoming (such as education). This resonates with the generative quality of “good” thought shapers (Maiese and Hanna, 2019). What discourages hope is distance from primary experience, isolation from other viewpoints and possibilities, and a self-fulfilling, self-closing (positive feedback loop) of despair in a community. This underscores the constrictive side of “bad” thought shapers.

To begin to explore these hypotheses, we follow Reed’s exhortation to host real conversations with those outside of academia (1996, 150–151). Who does not have primary experiential knowledge of hope, or its lack? While we could have likely interviewed (and would like to interview) just about anyone we happen to run into, for an initial investigation we chose to focus on expecting parents, a prominently salient population in the lifeworld of first and second author.

## Parent-guardian hope practices: A qualitative study

The planet is warming, a pandemic is raging, political tensions are ever-escalating, and yet, for now, for some, the world keeps turning. Consumption, production, and exploitation go on and so many of us remain stuck, complicit. Joy and good struggle are also part of life. Modernity is a site of perpetual contradiction (Machado de Oliveira, 2021). Life doing as it does, a few months ago, many of (first and second author’s) friends and family members were about to have a first or second child, and we wanted to ask them about their experiences of hope in this context. Is having a child now an act of hope, or of denial? Philosopher Joan Woolfrey argues that hope for a desirable future is the most important disposition to instill in children, and this hope must come from parents who genuinely experience it (2016, 132–133). Following from our theoretical work, we are highly motivated to make hope a topic of open conversation with those who are close and important parts of our lives, in hopes of building a community of action and hopeful practices.

We hypothesize that hope is a salient feature of pregnancy (as well as parenting) as it is a time marked by expectation and uncertainty on many timescales (the pregnancy itself, labor and birth, postpartum, caring for a newborn, raising a child). We note that the participants we interviewed wanted the pregnancies in question. While we do not have data to support this, we find it reasonable to think that persons experiencing unwanted pregnancy would showcase different dynamics of hope relating to that pregnancy (see text footnote 2). In calling for a more inclusive phenomenology of pregnancy that would attend at least to rejected and denied pregnancies, philosopher Caroline Lundquist argues that although pregnant people “may experience similar phenomena during their time as pregnant subjects, something is lost in the assumption that their lived experiences are qualitatively similar. Where they undergo analogous biological processes, for example, the willing and unwilling pregnant subjects yet describe their experiences in very different terms” (Lundquist, 2008, 140). Recent enactive treatment of pregnancy highlights the open tension of intertwined agencies, requiring participating and letting-go, that takes place within the pregnant parent’s own body in its relation to the developing fetus (Quintero and De Jaegher, 2020). While we suggest that hope is what refrains situations toward greater possibility, it is not clear that a “let it be and see what happens” stance is appropriate or sufficient in cases of unwanted pregnancy, in the absence of real material options for various agential responses. For hope to act as a generative thought-shaper, it must not become ideological or pre-determining. In fact, our theoretical message, corroborated by our findings below, is that hope discovers possibilities through reframing, but hopers require intersubjective support and an experience that there is more to know in order to enact hoping.

The study below looks only at a small, single facet of the experiential complex that is pregnancy and parenting. In the conclusion we address further studies that would follow directly on this work, both in terms of pregnant/guardian experience and in terms of enacting hope more broadly. We employ qualitative methodology to pose an open-ended, exploratory research question: how do expecting parents experience and enact hope? Our intent is to apply an enactive-ecological lens in this exploration, and to center voices of non-academics in our lifeworld who, due to their life stage and the particular existential weight of responsibility for lives to come, we submit are nonetheless “hope experts.”

### Study design and method

Employing a specific method of descriptive-interpretive qualitative research (Elliott and Timulak, 2021), reflective lifeworld research (Dahlberg et al., 2007), our investigation took the form of open-dialog interviews, conducted over zoom in January–March 2022, following institutional review board approval secured in late December 2021. Through email or by text message, (first author) invited participants, all of whom are friends or family members expecting or recently expecting to have a baby, to discuss their current experiences of hope, in an approximately 1-h conversation scheduled according to their convenience. Participants reviewed and signed an informed consent form in advance of the interview; this and the initial invitation offered a broad introduction to the purpose of the study. Each interview began with a brief reminder of that topic, stating the first author’s personal as well as academic interest in the experiences and viewpoints of the participants.

Following the idea of interviewing as “bridling,” the conversations consisted of a focused yet spontaneous, responsive sequencing of follow-up and new lines of questioning led by the interviewee(s) (Dahlberg et al., 2007). No set questions were used, though (first author) guided participants to consider their experiences of hopefulness (or its lack) on some of the different timescales mentioned above. “Researching with the practices of friendship means that although we employ traditional forms of data gathering (e.g., participant observation, systematic note taking, and informal and formal interviewing), our primary procedures are those we use to build and sustain friendship: conversation, everyday involvement, compassion, giving, and vulnerability” (Tillmann-Healy, 2003, 734).

All interviews were conducted over zoom and ranged from 30 to 60 min in length. The first author took notes during the interviews as part of data collection. Audio files from the interviews were reviewed and transcribed by the authors. The authors iteratively reviewed the text transcripts to produce thematic memos: after judging relevance in the data, we delineated meaning units, “…the smallest units of data (that are) complete thoughts capable of standing alone and communicating a message (meaning) relevant to one or more of the study’s research questions” (Elliott and Timulak, 2021, 46), and grouped them into themes or categories. Further analytical steps are described below.

### Participants

In presenting participant data, we proceed with particular care in attempting to strike a balance between a case study approach that preserves the depth and specificity of life histories and positioning and an aggregate approach that protects participant identity and shows trends across differences. Demographic information can be an objectifying and reductive way of presenting human beings, and particularly when working with a convenience sample, threatens to overdetermine the uniqueness of lived experience. At the same time, we have noted that positionality (e.g., race, education, class, etc.) may be particularly relevant to the external circumstances that afford or block hope.

First author interviewed parents, guardians, and parents-to-be from five couples; in one interview one parent/guardian did not join.^10^^11^ Of the nine participants interviewed, eight have college degrees, and five of those eight have advanced degrees. Three couples are presently in the middle-income tier and two are presently in the upper-income tier, based on location, income, and number of people in the household.^12^ Differences between participants include current location (three different regions of the USA and in rural, urban, and suburban settings), personal history of childbirth and parenting, and racial diversity in the partnership and in the home. One set of interviewees was expecting their first child. Another set had experienced child loss and child estrangement, and are considering the possibility of trying to become pregnant again. The other sets of interviewees each had one child (their own or in their care), ranging in age from 2 to 8 years.

Brewis (2014) carefully details a number of possible methodological and ethical concerns that may arise when doing research with friend-participants. For one, the researcher holds an unequal position throughout the interaction (Dahlberg et al., 2007), and the researcher moves private moments into a public realm (Brewis, 2014). Participants may forget in the course of the interview that the interviewer acts as researcher as well as friend and share more than they would in a conversation with a stranger researcher. Just as it is hard to predict the turns that an intense, sensitive conversation may take, or what emotional effects it may bring, it is impossible to predict how it will feel for participants to see their stories or responses in print, even if anonymized (ibid). We have sought to minimize possible harm to participants by engaging in an open and transparent data collection process that assures confidentiality and respondent validation. Participants were told from the start that their participation is voluntary and that they can end the interview at any point and can withdraw their participation/responses at any point. All participants were provided with an opportunity to first review, redact, and reflect on the transcripts produced from the interview, and later, provided with an opportunity to review and edit a late-stage draft of this manuscript.

### Analysis and results

Analysis of the data included multiple sessions of review of interview transcripts, interview notes, researcher memos, researcher meeting notes, and follow-up communications with participants. Analysis ran simultaneously with data collection, as interviews were spread out over a 2-month period. We approach the data from particular subject positions. The first author is a parent of two children, has had three known pregnancies, is married, is white, holds a Ph.D., lives comfortably, and is anxious about and implicated in the global crises of climate, racism, fascism, and greed. The second author is a MENA American male, parent of two children, married, holds a Ph.D., presently lives in comfort, and is a radical interdisciplinary philosopher. Third author is an African-American male, in his last semester of undergraduate studies with a major in Moral Psychology, an interest in phenomenology, phenomenological lifeworld research as a means to exploring individual lifeworlds as they are shaped by experiences as well as shared utterances, and as a means to understanding the conditions necessary for hoping. All authors have a background in embodied cognition theory and moral psychology.

The overall aim of lifeworld research is to describe and make comprehensible the lived world in a way that expands our understanding of human being and experience (Dahlberg et al., 2007, 37). This research methodology draws on the philosophical tradition of phenomenology. This is intended to be a beginning, a process of inquiry that gathers continued conversation around complex issues, in this case as a means to figuring out in which aspects of the lifeworld conditions for hope can be found.

Drawing on the foregoing theoretical reflections on enactive and ecological perspectives on hope, we were particularly interested to see how participants talked about hope. What is said (and how, and in what context) is at once a reflection of lifeworld utterances, of personal histories of incorporating and incarnating utterances, and of affordances of or impediments to agency. Yet, following our technical use of “linguistic” as in “linguistic bodies,” we also attend in particular to how participants responded to each other and to the unfolding conversation to make sense of hope. Our hypotheses hold that linguistic practices of “encountering the virtuality of the self,” such as de-centering, reframing, co-authoring, and sharing agency enable hoping as a paradoxical practice of staying in participation while letting go, while linguistic practices of overdetermination, labeling, fear-mongering, or other forms of “linguistic enclosure” limit hope. In interview conversations, the first author remained as open as possible to the topics and turns presented by participants, who were not told about specific theoretical commitments or hypotheses until the debriefing.

After clustering and coding interview meaning units (as discussed above), we generated themes of not knowing, transcending hardship, resources/privilege, trust in social progress, community, climate change, and a process description of reframing. We then checked to see “how representative the categories (themes) are within the sample of informants,” following Elliot and Timulak in their view that “the point of enumerating categories is not to emulate quantitative or positivist research but rather to provide a heuristic that interprets the meaning of the category within the sample, thus providing extra information and contributing to the transparency of the analysis. …it can be useful to characterize categories broadly in frequency terms that speak directly to the issue of generalizability” (Elliott and Timulak, 2021, 59–61). Following their example, we applied enumeration labels of “general” (more than 80% of participants expressed this theme), “typical” (more than 50%), “variant” (at least two), and “unique” (only one) (see Table 1).

**TABLE 1:** Enumeration of themes.

	Number of participants	Enumeration category
Not knowing	9/9	General
Transcending hardship	9/9	General
Resources/privilege	6/9	Typical
Trust in social progress	4/9	Variant
Reframing (process view)	5/9	Typical
Community	4/9	Variant
Climate change	4/9	Variant
Distrust in social progress	1/9	Unique

What emerges as most salient to participants when talking about hope are experiences of not knowing and experiences of hardship. We find it interesting to contrast this with a common moral psychological formula for hope of “desire + uncertainty of outcome.” Unknowing is by far the most consistently and commonly found expression from the parents interviewed. Desires (hopes for a particular object) as such were much less frequently and directly named. Across all interviews, we found instances of “I hope that,” “hope for,” and “hope (something happens)” constructions (for example, one participant said “I hope the baby sleeps” and another said “I hope to be a great parent”), but these were not the primary mode in which participants discussed hope. More common was the use of “hopeful;” we interpret this to be the expression of a stance in which participants find themselves. The greatest proportion of these uses occurred in an interview that had the most frequent mentions of “hard.” The pattern in this participant’s discourse was to speak of something being difficult and then conclude with the assurance, sometimes dubious and sometimes more confident, that she was hopeful, in spite of the clear reality and past history of hardship. Another frequent way transcending hardship was expressed was in resistance to anxiety and depression, or simply in detailing the more difficult aspects of pregnancy or early parenting as having passed.

Yet the fact that enacting hope is a response to unknowing and difficulty is, on balance, to be expected, given both everyday and academic knowledge of hope. That meaning units pertaining to resources and privilege, and those demonstrating reframing, were typical in our sample is perhaps more telling, as these potentially shed new light on hoping phenomena. We discuss these further, below.

#### “This faith in like really having no idea”

We offer a selection of meaning units from the interviews to show a range of contexts and ways that participants expressed not knowing. As meaning units can pertain to multiple themes, some of these also anticipate observations we share in the following section.^13^

(1)“…I don’t think I have a vision of blackness in the future… I think I do like major societal aspects of society like technology and race and you know environment and health things are all pretty bleak, but I’ve got a lot of privilege so a lot of that doesn’t impact me and so I can just focus on like growing a family and building a life and I’m hopeful but that I think otherwise I wouldn’t have considered to have another kid um so to me yeah I don’t know.”

(2)“…like I really think we’re going to be Madmaxing it in like 20 years. You know what I mean like we’re going to be wearing like furs and stuff… I’m cool right now but also like that’ll probably be okay… like I don’t know, we’ll find a commune. I don’t know.”

(3) “Oh, yeah, I don’t know that I’m expecting much. Actually, more just thought, just anticipation. Right? Like, you know, and I think I know enough to know that anything that I think I know is wrong. So I shouldn’t really do anything other than be open to, you know, the experiences that we’re going to have.”

(4) “So I mean, I think so that like, you know, the hard part was the sleep, the unknown. So what do you do? And then all of that, while trying to manage keeping life together with no support, and then the outside, like, what is actually happening? Are we safe? And is my child safe? Can we go to the grocery store? Should we go to the grocery store? All that.”

(5) “I think that that can be really hard, especially from different experiences you go through, um, and so and that’s why you know, um, it’s, it’s gonna be hard like, I don’t know what he’s going to experience or anything like that. And it’s like, I can’t prepare him for any of that.”

(6) “Well, my first thought is like, I’m nervous about meeting him. Because I don’t know, like what he knows, you know? Yeah. So I’m just nervous about it. Like my meeting with my dad. The first time was horrible, you know, so.”

(7) “Yeah I’m not really optimistic that–I don’t know, that’s the thing, right, I also have this faith in like really having no idea… Like I’m about to go into this birth and I have no idea what’s gonna happen and I’m frightened in some ways and the only like balm I can get from it is like really knowing that I have no idea. Like it’s not even hoping that it will be good but like being just like whatever happens I’m going to have to make the best of it basically and that’s how I feel about the future too like maybe we’ll be living in a weird commune and eating squirrels and maybe we’ll find joy in that? I don’t know.”

In these excerpts one sees the complexity and polysemy of the use of “I don’t know” and similar expressions. While expressing uncertainty toward the future in the sense of what is going to happen, the quotations (1, 2, and 7) demonstrate usage to mark the epistemic stance one takes toward what one is saying (“hedging”). Especially at the end of a turn, “I don’t know” leaves open the relationship between the speaker and the proposition they have made. Research indicates that “you know” and “right” are similar epistemic markers as they seek intersubjective affirmation rather than framing what is said as certain (e.g., Landgrebe, 2012); certain participants used these expressions frequently. Participants made pragmatic, interaction-management uses of “I don’t know” expressions in relation to not knowing what the other interlocutors know (“I don’t know if I told you…” “I don’t know if you know that…”). We also found expressions of not knowing in the present: not knowing something about one’s own child, e.g., “I have no idea where he got that from,” and a general state of unknowing that pervades parenthood in the form of self-judgment of questionable expertise: “I have no idea what I’m doing,” and see quotation (4).

Most interesting to us are the statements of not knowing what is going to happen. Participants expressed this in regards to specific examples, like what it would be like to see one’s child from whom one has been separated for some time [quotation (6)], or what it will be like for one’s child of color to experience growing up with white parents and a white sibling [quotation (5)]. Some expressed broad difficulty in imagining the more distant future with confidence or clarity, and resorted to dystopian tropes (i.e., MadMax) [quotations (1), (2), and (7)]. In all of these instances, anxiety was present or co-expressed (e.g., “I am terrified”; “I’m nervous about it”), but immediately countered with a resolve of personal commitment to being responsible inside of that experience and potential difficulty as it unfolded (e.g., “whatever happens I’m going to have to make the best of it”; “I’m still like, open to it, you know, like, I’m not gonna run from it.”).

#### Additional themes

A sense of resolve as an existential stance (De Haan, 2020) in the face of an uncertain future pervaded a number of interviews, constituting a theme of hoping as transcending hardship.

(8) “I feel like there’s like there’s things that I’m not worried that the world is going to be a worse place, there are certain things where like, we should really believe everyone should be much more focused on climate change than we are because that’s just like an existential worry that I know my kids and my kids’ kids are going to have to deal with now. And I wasn’t worried about it in the same way before, but I was saying, like, even if the world is worse off than we are going forward, I feel like our little unit and what we can do, like I have confidence that outside might be burning. But you know, we can still provide like a stable, safe, happy, loving environment no matter what’s kind of happening outside. And that’s sort of like, that’s gotten me through some of my existential angst toward particularly like, you know, mid COVID waves of what’s actually happening in all of that.”

(9) “It would be paralyzing otherwise, right? Because like, you’ve got these things that are they’re always looming over you like, yeah, climate change will absolutely probably is a problem, right, and we absolutely have a crisis to deal with about it. We’re not gonna deal with it in a way that does, that erases all of the irreparable harm, right? Like, we’re gonna have to live with that harm going forward. But it’s kind of the same way that you know, you have wounded bodies or wounded cultures, and you can live going forward with that stuff, right? It’s not like, the end of all things. And we’re gonna be like, the meteor or something like that instead, right?”

We notice that the possibility of transcending hardship is frequently linked to resources and privilege, as in quotations (1 and 8) above, and here:

(10) “Like if [baby] is healthy, and you’re healthy, like we are just set, like, any other problem we can just deal with, we can throw money here, whatever, like it’ll be, it’ll be fine. Right? Like, we are in like, largely champagne problem territory, I think as long as we get to the outcomes of everybody’s healthy on the other side. And so that’s kind of my constant framing.”

(11) “And I think to like, knowing that we can kind of, we would like to transmit that to our [baby], and like, because I always see my so my parents have always been very active, like, they’ve always my dad runs still, you know, like, they still work out and stuff. And I think just seeing that, um, I kind of picked up on it, and I was always active. And so I would like that for [baby] as well and kind of give him those same resources. Or at least perspectives.”

At the same time as participants voiced a perhaps slightly qualified confidence in a broadly safe future for their children, several also indicated that they wanted something more than safety and survival.

(12) “…hope is more about I guess, like a level of optimism for the future, right? And maybe that you don’t have to be resilient. In that future scenario, or more importantly, like that [current child] does not have to experience being resilient in any type of extreme way for long durations?”

(13) “…you want them to be–and that’s what I’ve always told my nieces too, even when they were younger–I want you to be a better person than I am. And, or like to do better things or more things that you know, I’ve done…It’s not necessarily like, oh, my gosh, you have to go do this education or, you know, things like that, but it’s just like, I want you I just want you to be an overall like, well grounded, great person.”

(14) “My hope for [children whom the participant knows] is that they have an easier life than we had growing up. Like, and they have parents that are maybe more aware. Or even that they are willing to try different things that maybe we weren’t as kids.”

What the “better” would be for their children is left open, explicitly, in participants’ remarks:

(15) “Especially with [current child] like, like embracing possibility, right? It’s not that, like, I don’t hope that he becomes [redacted], or that he you know is wealthy or does anything else, like I have almost zero preconditions? You know, my, my hope, or my perspective on it is more that it’s about discovery, right?…And I think that, you know, that excitement is really what sparks you know, hopefulness, in terms of like, well, what’s he going to do?”

There was ambiguity in whether hope for the future must be linked to a modern notion of progress. As shown in Table 1, expressions of trust in the direction in which society is heading were variant (but nearly typical in the sample) while what the third author described as “confident distrust” in the same was frequently expressed by one participant, who at one point said,

(16) “I think we’re in a weird spot cuz we were raised with this like unlimited sense of hope that the boomers instilled in us that was like anything is possible because the economy is so good and like cuz our lives were better than our parents right that’s like their perspective was like their parents grew up in the depression and survived WW2 and the second half of the century was like honky dory by comparison, so they’re like oh this is great and you guys will be able to do it anyway, you can do anything you want, right, that’s what we were raised to think and I don’t think that’s true.”

Of note, those expressing trust did not discuss climate change at all, and were not in the highest income bracket. Another participant, after expressing extended concern about homophobia and racism on Facebook and in public schools, cautiously expressed trust in social progress:

(17) “You’re terrified when you get to the real world, you know, and they’re out there living their life, how are you going to? How are you gonna do in those situations, you know? …you’re hopeful that it will be great. And that, like this generation, and a lot of people that, you know, you’ve talked to and see, it’s going to make it better, however, yeah you can only do what’s in your control.”

Similarly,

(18) “And I think there’s, you know, lots of hope for how our world could go. I think that there’s more people that are becoming more open to all types of things. But I still think that there’s those negative things as well. And I think that’s what makes them feel hopeless.”

We note a significant theme of community. When asked about what participants do or have in their lives to turn to when they feel hopeless, the answer often indicated the role that other people play. Several participants spoke of their romantic life partnership as a source of hope:

(19) “…I worry a lot about our relationship and like, how is that going to change? And I’ve told him that so many times, and he’s always like, we’re gonna be fine. Like we, you know, we’ve been together 14 years, almost, I guess. Anyway so it’s like, he’s like, we’ve made it this far. Like, we’re gonna always be like, we’ll stick together, you know what I mean? …And that gives me a lot of hope too, not just for like, our family with [baby], but like us as a couple. And I think that’s really important not to lose sight of.”

(20) “But I guess other like, just for inspirations, I think we both kind of rely on each other? A lot, too. But we’ve been together long enough that like, more than half my life, like, we’ve known each other half our lives now. Yes, which is crazy.”

Notice that one hope-shaping practice (among many) a partner provides is that of reframing hopeless utterances. A partner gives assurance, structure, and inspiration [quotations (19) and (20)] and can also present the shared situation in a different light. Here is an extended example:

(21) “So like 3 days ago I was like, I don’t even want a baby. I don’t want a baby. I don’t want to go through labor. I don’t want to be taking care of an infant, don’t want another kid, why are we doing this [laughs], this is a mistake. I’m so, this is fucked up just kind of like in this feeling of like, a lot of it has to do with um the labor itself being like ah, fuck, like come on, I just want to sleep and be by myself. [Current child] just went to school this year, we just got out of like 18 months of like no school and no work for me so like all mama all the time parenting, and it was hard you know it was really hard, but anyway I was like oh man I don’t want to do this and I was feeling like really shitty about it, and then I got a text from [partner] that was like, “are you gonna have your lovely baby today?” And I was like awww it *is* gonna be a lovely baby! Aww it’s gonna be so nice, and just having that sort of like you know that little bit of uplifting energy from you know the person it affects the most [laughing].”

#### The role of community in utterance reframing

Regarding the cultivated capacity that linguistic bodies have to report, reflect, and reframe utterances, Di Paolo, Cuffari, and De Jaegher write, “The power of enacting dialogs through the use of reported utterances makes them simultaneously transformative of both social relations and individual minds” (2018, 190–191). Within the deeply shared lifeworld of a partnership or a family, the other doesn’t need to repeat the exact wording or even tone of one’s utterance in order to refer to it. In fact, it is the complete contrast of tone that this participant’s partner’s text enacts that makes the difference. The participant’s attitude toward the pregnancy, upcoming childbirth, and parenting demands had become somewhat despairing and negative. This perspective could have been enacted by self-directed utterances, outward expressions, or a combination of the two. The partner’s text message at once refers to the potentially burdensome event on their shared horizon and presents it newly, as a sweet, uplifting thing that one looks forward to. More specifically, the text message puts the focus on the baby that is coming, names it lovely, evokes a desiring expectation to hold and embrace this new life. The reminder that other perspectives are possible, and possibly true, that the quality of the future is not predetermined or foreclosed, affords hope. As the speaker of quotation (21) went on to say, “But that really changed, it was like before I got that text I was like (negative noise/gesture) and after I was like aww no it is gonna be really nice. And so I feel the sort of like the community thing I think is a big part of it. Hope is like, I don’t know, it’s a vibe and you can spread it.”

The latent ability of all dialogs to “go meta” and become about themselves through recursive uses of utterances is what makes them “powerful active instruments for making explicit and questioning the normative structures that frame (dialogs) and other social activities” (Di Paolo et al., 2018, 190). It also makes dialogs perpetually “susceptible to reaffirming their frames uncritically when utterances and participants circulate in closed circles, reifying local norms as established universal truths” (ibid). Quotation (21) shows that close circles nevertheless harbor multitudes of viewpoints and evaluative, existential possibilities for sense-making, so long as members of the family, partnership, or community can remain open to other voices. When one can stay in the place of not-knowing, new interpretations have a chance to enter the flow of self-directed utterances and get a grip there. Especially in a close community, the other doesn’t have to report your utterance back to you explicitly, given the depth of the shared common ground. At the same time, historical habits of attending to and caring about the utterances of a particular author can imbue them with a kind of authority that can cut through one’s own patterning. In this way, individual and community sense-making intertwine, and not knowing can dance with knowing. We do not know what the future holds, but we know each other.

Groups may continue to dialectically refine knowledge of their shared lifeworld through interactions with each other, and this experience of not-knowing-together enables groups to enact a shared hopeful, open stance toward what is coming. One participant described a plurivocal community as a positive environment for child-rearing, saying,

(22) “I think a family of choice is sometimes more important than your blood, biological family, right? I don’t think everybody wants it. But I think there are some parents, you only want your children to learn your way. But I think people that are like, I want multiple influences on my child, hopefully all positive, so that they can become the best person that they can be. And I think that’s beautiful. [Partner] and I talk about family of choice often, like, you know, how you have some friends that like you love you care about, they’re not necessarily people that you want around your kid all the time, or even like family members that you might love. But you know, that might not be the most important. …I think there’s hope in having that flexibility.”

Plurivocality is an important condition possible on a variety of scales, including, ideally, practices of listening and granting authority to voices quite far from one’s own family or community. To again quote Reed, “…the most important component available to us for producing hope” is “learning to respect the experience of others” (Reed, 1996, 156). A precondition of hope, then, is maintaining an ability to participate-while-not-knowing, to engage without having or demonstrating expertise. Developing the conditions necessary for hope requires that we continually enact the practice of making room for multiple voices. These are also the conditions necessary for solidarity (Fourlas, 2022). This practice enables us to make the natural attitudes that define our personal and communal lifeworlds more salient to us and others. In turn, this awareness enables us to encounter the virtuality of the self, and so frees us for intentional commitment to our relationships and responsibilities.

## Discussion

In this exploratory lifeworld study, we distilled some core meanings that can be further investigated. We hypothesized that encountering the virtuality of the self through a dialogic encounter is “good” for hope or is a practice that enacts hope. This is rather abstract and leaves open how one goes about such encountering; however, we do see some corroboration of this view in the interview data and in participant follow up reflections. When asked to talk about hope in light of upcoming or recent births, participants expressed their thoughts and experiences largely in terms of their epistemic relation of not knowing. Not knowing is how they made sense of hope and in some cases was explicitly what strengthened their affective experience of hope. Facing the future is a confrontation with the virtuality of the self; who one is going to be remains an unwritten story. Parenting is a practice of participating in something uneven and unknown, an on-going shaping of another person’s becoming. Expressing uncertainty, reflectively dwelling in it, even, is hopeful insofar as it holds room open for (or at least does not foreclose) possibility in that becoming. We saw as well that practices in a partnership or family of making room for multiple voices, perspectives, and evaluations afford rescue from despair, not only in the sense of interrupting or countering negativity, but also in that practicing plurivocality can free us from the isolation of our own routines of sense making.

While it would take us beyond the scope of the present paper to unpack the claim properly, the praxis of hope that we sketch here may stand in some tension with a predictive brain model even as reframed in eco-enactive accounts that hold that agents enjoy new or surprising things because “when an agent succeeds in reducing error at a faster than expected rate (or recognizes the opportunity to do so) this feels good. It is thus not uncertainty reduction alone that the agent cares about, but also the rate at which uncertainty is being reduced” (Kiverstein et al., 2019). Perhaps hope is a fraught experience phenomenologically because by definition it lacks the comfort of reducing uncertainty. This proposal follows the logic of entanglement of bodily domains and personal and sub-personal processes (Di Paolo et al., 2018) but requires further investigation to see if the existential domain of hoping can be connected to brain-body perceptual dynamics.

At the same time, we present our work in resonance with the ecological-enactive idea of thought-shaping or mind-shaping (Maiese, 2022). The mindshaping framework helpfully highlights linguistic bodies’ vulnerability to “maladaptive manipulation by mental institutions” (Krueger and Maiese, 2018, 15). If hope is an objective feature of the environment, languaging practices, as constitutive of human environments, will be part of what safeguards or threatens hope. In dialog and in stories we can find new meanings or stubbornly hang onto dying ones (Machado de Oliveira, 2021). We can circulate anxieties (looking at you, 24-h cable news networks) or wake up to the fact that at least, in speaking together, we are not alone; there may yet be things in the environment, in each other, left to discover. Yet languaging remains complexly imbricated with other realities of a shared environment or lifeworld, such as class and material circumstances.^14^

One relevant, delimiting aspect of our study, in addition to the small sample size, is the relative privilege of the participants and of the lifeworld in which they and we as authors co-exist. This is especially salient in facing the question of the global climate crisis. For a few participants, the climate crisis showed up as a worry, but not a fully dominating one, not one that rules out having children or overshadows their planning. Climate change discourse is an irritating background of their lives, as one participant describes, “the persistent like buzzing threat, that’s always there.” The story these participants tell has the vague shape of being prepared or being in a good position for when this issue becomes more center-stage. Others did not address the climate crisis at all, indicating that the lifeworld concerns that worry them or, in other words, elicit the work of their hopeful participation, relate to racial justice and the question of a peaceful society. While these concerns are entangled undeniably with the climate crisis, we tentatively submit that we and participants exist in a sort of temporal privilege in addition to a material privilege related to the disastrous effects of climate change (among other global crises). We can “dodge the bullet,” at least for now, likely with another decade or two of relative protection. We work toward graduation or tenure, save for the future, and pursue art and knowledge-creation. In short, we are not dealing with the worst effects, yet, and so we do not feel that we have to, yet.

The “yet” is there, though, and highlights the uncomfortable precarity of all privilege: it is not stable and will not last. At some moments we may realize that it should not last. The “yet” is a pressing question for parents or caregivers of privileged positioning, because it underlines an absence of knowledge we may expect to be highly salient, indeed, life-determining for our children. But the “yet” still marks a remove from millions of others who are in an “already” relation to crisis. In the particular lifeworld that we broadly share with our participants (capitalist, colonialist-modern, educated, USA), having children and persevering toward a good life for them can consist in practices of radical hope [in Lear’s (2006) sense], and it can consist in indulgence and denial that actively harms others.

One way to relate to this “yet” is suggested in Vanessa Machado de Oliveira’s writing about moribund modernity. She points to a Brazilian saying “that in a flood situation, it is only when the water reaches people’s hips that it becomes possible for them to swim. In other words, we might only be able to learn to swim–that is, to exist differently–once we have no other choice. People’s priorities are bound to the level of the water around them” (2021, 38). We take this as an important insight on its own, and an invitation to have compassion for struggle as a variegated process that need not and cannot look the same everywhere and for everyone. Ankle-deep water for an adult could be knee-deep for their child. Perhaps this can serve as motivation to “create spaces in which new habits of behavior and attention can be developed” (Maiese, 2022, 14).

Perhaps a way to enact hope inside of a privileged (anxious) temporal relation to crises is to participate intentionally and see oneself as a participant in two ways. We note, with Fred Cummins, “Participation is a voluntary surrendering of autonomy. It is not a mechanical act” (Cummins, 2019). A distantly temporal relation is still a relation; conducting oneself toward the future is always done in the present (see Di Paolo (2021)). If one acts in the present as a participant in the crisis, there can be relief, an easing of a tension, that allows room for hope. To be clear: being in a privileged position now means one is a participant in the present crisis of another. Shining light on this present underside of privilege can perhaps facilitate hope in the same manner as realizing that there is more to find out, more than one’s own perspective. The aim is to enable hope “to weave relationships and movements in the present–the very textures that futures are made of. Whatever happens ‘then’ depends more on the quality of relationships in the ‘now’ than on the accuracy or appeal of images of the future that one projects as a way forward” (Machado de Oliveira, 2021, 37).

Present-oriented participation can take the form of recognizing, appreciating, and engaging with the “otherness” of children’s perspectives. Children are a continual source of difference in perspective through which their caregivers can practice listening and being open to reframing (a condition of hope). Perhaps unlike in previous generations, Western/Northern children today may have less privilege, at least temporally, than their parents, their existential horizon shorter. This is a deep asymmetry, in addition to the basic epistemic asymmetry that divides parents and children (Overall, 2016). In their interactions, children and caregivers alike have deep need, and opportunity, to foster hope across generational differences. As philosopher and activist Báyò Akómoláfé writes, “…perhaps the most challenging practice… is to meet our children anew, seeing them as teachers and elders. By blurring the generational walls made firm by a schooled society and its addictions with class and hierarchy, we are learning to think of our children as spiritual companions, as ethnographic guides into the terrain cleaved open by our dangerous dissertations of distance and supremacy. I am learning a sense of the divine in hanging out–or failing to hang out–with my children. I am learning that being a father in a time when making kind is so problematic that we are invited to make kin instead can be a sanctuary” (Akómoláfé, 2019). We see here a picture of parenting as a hope practice: letting others lead, letting go of the widely accepted idea that parents must always be the teachers, the wise ones. Paradoxically, if privileged parents teach our children to live as we do, to function well in ‘‘our’’ society, we are in a sense teaching them how to usher in the end of the living world. ‘‘Bringing new life in,’’ as one participant described her situation as an expectant mother, is a mighty responsibility, and a chance to be undone and remade.^15^ It is particularly hard to hold on to such a counterintuitive perspective, however, when caregivers are constantly, unconsciously (and consciously) teaching children about the world. Stories, songs, or rhymes–interactional routines–are a rich source of language acquisition and are also a source of culture and value acquisition (Bruner, 1985). They are a training ground for how a community will “sing the world,” to borrow from Merleau-Ponty. The use of “I” in daily speech and thought, the practice of speaking as a single subject, and the orientation to a “straight-ahead” future are deeply ingrained habits of linguistic closure for many colonial-modern adults. Fred Cummins draws our attention to the fact that many of the most affectively powerful group experiences consist in joint speech, for example prayer, protest, and song (Cummins, 2019). While we can only here make a small suggestion, joint speech practices and imaginative play with children may be some ways to reduce our grip on our selves and our ways of doing things, in favor of a participatory practice of hoping, such as Marcel indicates when he writes, “I would be very tempted to say that all hope is at bottom choral” (Marcel, 1973, 143, as cited in Michener (2020), 85). Communities and families practice hope when they join and reframe each other’s expressions to encompass difference and moreness.

## Conclusion

In this paper we presented a theoretical perspective on hope as a sense-making activity of linguistic bodies. Sense-making is never neutral; intentional living things are always toward the environment or world in some way or another, in pursuit of some values (Hodges, 2007, 2022; Colombetti, 2014). When what creatures like ourselves are “being toward” is uncertain, the stance we take in sense-making may be hoping. We sought to operationalize hoping as a community-enabled praxis of resisting and reframing utterances that pronounce outcomes or judgments definitively. This hypothesis builds on existential, phenomenological, and enactive approaches to hope (e.g., Marcel, Lear, Thompson). We reported results from an exploratory qualitative study consisting in open-ended interviewing of new and expecting parents/guardians with whom we share a lifeworld (i.e., friends and family members). We analyzed this data through the lens of our theory using reflective lifeworld and descriptive-interpretive methods. We now return to some of our opening discussions of hope scholarship in light of our findings.

At the start we noted four unresolved tensions in the literature: (1) whether or not “real” hope has an object, (2) whether hoping is a virtuous activity or basic cognitive activity, (3) whether or not hope is contingent upon external circumstance (as opposed to simply being a matter of will), and (4) whether hope is politically desirable.

We make a move familiar to enactivists in suggesting that the first three tensions are best seen as moments ripe for dialectical transcendence, rather than as static oppositions. Hoping is a stance and a practice; it seems reasonable to us that hoping can be enacted in reference to a more or less specific object or situation. Put differently, since hoping is an intentional (sense-making) activity, it cannot be completely without object. The fusion of ecological psychology and enactivism in recent years further pushes back on the common conception of “object” and “organism” as individual and opposing systems in favor of something like an umwelt or “shared history of organism and environment” that itself gives rise to things that function like objects, stimuli, or affordances for organisms with a certain kind of capacity in a certain kind of environment (Fuchs, 2018, 128–129). If hoping is not disembodied, it is also not severed from material environment nor existential situation, and so is “about” something, even if that something is more or less well-defined.

It follows that resources and material conditions matter, which indicates a response to the third tension above. We take the same view as Edward Reed: hope is objective in the sense that its conditions are out there to be found (or they are not; the situation is “hopeless” as we say) in our encounters with the world, and our collaboration with such conditions is accompanied by hope as an aspect of experience. There is an unresolved tension in the psychological view that pathways thinking is tantamount to hoping and that anyone can engage in this sort of thought process. We suggest that pathways thinking is a species of self-directed utterance reframing. Such reframing in turn depends on real material conditions of possibility. Specifically, a community-supported habit of reframing supports individuals and families to bring a fuller range of possibilities (and material, concrete resources/strategies/steps) into view. Therefore, without conditions for multiple possible actions and for agency, and without a trained ability to reframe how one speaks to one’s self about one’s situation (a training that relies on education, community reinforcement, and that is frequently enacted in intersubjective dialog), hoping is not afforded. Hoping depends on these conditions being in place, and hence is not available to everyone all the time or to the same degree. We believe the deaths of despair case studies exemplify what happens when such conditions are absent or deficient.

Regarding the second tension, we suggest that hope, while virtuous (in the sense of being morally desirable and requiring practice and cultivation), is also typical in human cognition. On the view we present, the mechanisms of hope are the mechanisms of utterance management and perspective-taking that are constitutive of being and sense-making for linguistic bodies. The know-how of hoping is a know-how of not knowing. By this we mean, a know-how of interrupting the closure of the “open loops” by which humans anticipate what’s coming next in a “functional cycle,” be that one of sensorimotor coupling with an environment or existential sense-making (e.g., making a decision or judgment) regarding one’s life (see Fuchs (2018), 129). Hoping suspends such closure, perhaps by holding silent, by offering an alternative framing, or by shifting attention. These mechanisms require further study, especially insofar as they may span or entangle sub-personal and personal bodily domains.

A further avenue of research would investigate the affective conditions (also likely typical) that spur enacting hope. We suggest that one contender is ambivalence, or a feeling at once of difficulty and refusal to rest with that difficulty. The sort of reframing that we see in our participants’ discourse demonstrates this sort of multiplicity of feeling, for example:

(23) “For optimism, well, you know, things can be both bad and better. Right? Like, you think of it as in, if [redacted] ends up in the NICU, right, and we have to be there monitoring every vital sign for months, like [redacted] did with their child, you know, every single day, like objectively bad, like [redacted] is in the NICU, this is bad. But he was also better every single day. And now he’s home. Right? And so like, I think keeping the framing of like, yeah, like things are bad. But you know, you can still be better in that context is a good way to think about it.”

We take this sort of thinking to be emblematic of West’s notion of “struggle against the evidence,” as the perspective reported in quotation (23) counters an “objective” description with another frame, one that makes space for a positive interpretation in addition to the negative one. While more work is needed to make such a claim, we take examples like these to indicate that human cognition is not solely and perhaps not ideally rooted in prediction, but in a kind of refusal to predict a single outcome or assign a single value to an expected outcome. We suggest that enacting hope is rooted in basic valuing and sense-making mechanisms, but potentially enables people to practice a virtuous mode of participation insofar as people, in hope, let go of the goals of optimal grip and reduced uncertainty. In other words, we suggest that hoping is a basic cognitive-affective activity, which requires a thinking against knowledge. That kind of thinking (counterfactual, etc.) is possible and perhaps unremarkable (if the right conditions are in place), but nonetheless is potentially transformative because it builds virtues such as courage (Lear, 2006). The information age perhaps puts higher cognitive and moral demands on hopers. We are drowning in what is known with less room for what is not known, and less opportunity for one-to-one exchanges with the sources of the daily information flow. A focus on what is known (the planet is warming, dependency on fossil fuel is ruining lives in the short and long term, e.g.) that forgets the moreness of what is not known is a sure recipe for despair. We need to keep space for this kind of not-knowing, and we do find this work of maintaining openness to be politically desirable.

The present study was small and exploratory, and speaks from a particular lifeworld perspective. Further studies using other methodologies should be conducted in the future to confirm our claims. The experience of hope in cases of unwanted pregnancy seems to us to be pressing and salient as a follow-up investigation, as do various investigations of interactional strategies that help bring children into the practice of hoping as not-knowing.

## Data availability statement

The raw data supporting the conclusions of this article will be made available by the authors, without undue reservation.

## Ethics statement

This study was reviewed and approved by the Institutional Review Board at Franklin and Marshall College. Participants provided written consent to participate in the study.

## Author contributions

EC contributed writing to all sections, conducted interviews for data collection, contributed to data transcription, and led data analysis. GF wrote parts of Introduction, Analysis and results, and Discussion sections and assisted with data collection and analysis. MW contributed to project conceptualization, data transcription, and analysis and wrote parts of an enactivist and ecological approach to hope practices and Analysis and results sections. EC and GF revised the manuscript in light of reviewer feedback. All authors contributed to the article and approved the submitted version.

## Footnotes

1 The reader may be wondering what we mean by “languaging” and “linguistic bodies theory.” Hopefully this orientation will become clearer as the paper unfolds, exemplifying these terms. A brief primer in the meanwhile: As we know it and use it, “languaging” dates back to Maturana’s (1983) usage (in “What is it to see?”) and over 40 years is invoked in different ways to demarcate an enactivist approach to language as interactive behavior. In solo writings and works co-authored with Francisco Varela, Maturana examines language-as-languaging by focusing on “individual actions in their constitutive relational nature, as taking effect only through the actions of others. …languaging abilities are therefore coordinative abilities” (Raimondi, 2019, 20). Over the years, many scholars have commented on and developed Maturana’s usage, in particular (Bottineau, 2010, 2012; Stewart, 2010; Kravchenko, 2011; Thibault, 2011; Raimondi, 2014, 2019, 2021). Cuffari et al. (2015) and Di Paolo et al. (2018) offer an account of language, linguistic bodies theory, that begins with participatory sense-making (De Jaegher and Di Paolo, 2007), which, like Maturana’s (1978) idea of structural coupling, systematically accounts for the ways that interactors’ acting-perceiving behaviors and experiences provide the conditions of possibility for shared meaning in a co-created and co-managed domain. They aim to offer a “basic corporeal logic of what is involved in the activity of using and enacting language” (Di Paolo et al., 2018, 133). This is described briefly in Section “Sketching an enactive and ecologically informed approach to hope practices” of this paper. The enactive tradition is informed by pragmatics, pragmatism, phenomenology, and Anglo-American philosophy, while retaining connections to biology and cybernetics. Historical antecedents and touchstone reference points include Wittgenstein, Merleau-Ponty, Mead, Vygotsky, and Austin, as well as work in cognitive and interactional linguistics. Recent theoretical work continues to refine the approach and bring it into dialog with ecological psychological and enactive-ecological approaches to language, such as van den Herik (2019, 2021); Kiverstein and Rietveld (2021). Linguistic bodies theory is intended not only to be a philosophical theory, but an interdisciplinary research program. Empirical research in domains of gesture (Harrison, 2021) and dance (Welch, 2022) showcases how linguistic body analyses can work with behavioral data; we submit the present work as a contribution in this vein.

2 Though it is not the focus of the present study, given the political scene in the U.S.A. surrounding abortion today, it is pressing to note that hope can relate to pregnancy in myriad ways. One can hope not to become pregnant. If pregnant, one can hope to get out of that situation (in some states, possibly without any concrete plan for doing so). Following from the view we present here, a person carrying an unwanted pregnancy may find it particularly difficult to enact hope, given that there are few and highly contested discourses from which to appropriate utterances or examples of reframing utterances toward non-local possibilities. In other words, it may be challenging to convincingly tell oneself that one will have access to resources to care for an unwanted child that must be brought to term, or that parenting will be easier than one expects, or that one will receive compassionate and safe medical care to terminate the pregnancy, or be able to afford or access such a procedure.

3 Novels can misdirect us toward an over-focus on the main character as an individual; a more in-depth analysis of these stories would reveal the significant others and the situations that enable hope in these characters. Indeed, the tendency to an individualistic modality is precisely what the more hopeful of the existentialists, Camus in particular, were trying to push back against. Given that we are all already struck by the plague, that we are all Sisyphus, and thus thrown into a common condition, the question becomes one of creating meaning together, not further isolating ourselves to a solitude where meaning is constrained at best.

4 Here, we are not dismissing emotion as a part of hope; rather, we are concerned with accounts of emotion that reduce phenomenon like hope to merely subjective feeling. For more about the enactive stance on emotion see, e.g., Colombetti (2011, 2014, 2017), Candiotto and De Jaegher (2021).

5 The authors note, rightly, that people of color in the USA and Black people in particular have harder lives and higher mortality rates, and, moreover, they too have been more recently affected by the deaths of despair trend as the opiate pandemic has reached Black populations (Case and Deaton, 2020).

6 In a chapter on pragmatism and hope, Stitzlein (2019, 105) quotes Rorty (2000, 3): “[S]ubstituting hope for knowledge, substituting the idea that the ability to be citizens of the full-fledged democracy which is yet to come, rather than the ability to grasp truth, is what is important about being human.”

7 Our use of “historical bodies” is meant to highlight the materiality of bodies as natural-cultural mashups. See McNally (2001), and text footnote 1.

8 This is well captured in Jonathan Lear’s telling of Plenty Coups stewarding of the end of the Crow way of life [Lear, 2006; and see Thompson’s (2021) discussion].

9 The linguistic relationality of the self has been well documented in several philosophical traditions, notably in the dialogical phenomenology of Buber (1970/1923) and Stawarska (2009).

10 “As I speak, I counter-interpellate the language that interpellates me to my place as speaker, which makes me what I am” (Lecercle, 2006, 145). This Marxist perspective on language as most rightly understood as a site of subjectivation is another possible way of understanding the closure of the “I” as an individuating act that nonetheless ties one to a place in a social relation and community and paradoxically overwrites the non-singular or non-static becoming of a living being who is and is not an individual, and whose use of a language (and its pronouns) both maintains and changes that shared form of praxis.

11 Regarding sample size, in reflective lifeworld research “the question of variation is more important than the question of number” (Dahlberg et al., 2007, 175). We have sought variation within a set of participants that is defined in terms of proximity to the first author. Furthermore, as it is “founded in phenomenology and hermeneutics, lifeworld research holds the ontological and epistemological idea that meanings are infinite, always expanding and extending themselves. Consequently, no meaning (data) saturation can exist” (ibid, 176).

12 Pew research calculator. https://www.pewresearch.org/fact-tank/2020/07/23/are-you-in-the-american-middle-class/. Accessed May 18, 2022.

13 Quotations were edited only to remove word repetition that did not contribute to meaning.

14 “Language involves materialism in the strictest sense in that it involves speaking bodies; and it involves a broader materialism…that of institutions and apparatuses, in that they produce discourses and speech acts” (Lecercle, 2006, 175). Language as praxis is constitutively historical, social, material, and political (Lecercle, 2006).

15 We find this insight applicable to non-parents and non-guardians as well. Transitional periods are opportunities to experience the virtuality of the self. Any one is (potentially) “bringing new life in” when engaging intentionally in transitional moments, or toward uncertain futures, or when forced into upheaval but able to stay present for it, etc.

## References

[B1] Akómoláfé B. (2019). *Making sanctuary: Hope, companionship, race and emergence in the anthropocene.* Available online at: https://www.bayoakomolafe.net/post/making-sanctuary-hope-companionship-race-and-emergence-in-the-anthropocene (accessed September 7, 2022).

[B2] Blöser C. Stahl T. (Eds.) (2019). *The moral psychology of hope.* Lanham, MD: Rowman & Littlefield Publishers.

[B3] Bottineau D. (2010). “Language and enaction,” in *Enaction: Toward a new paradigm for cognitive science*, eds Stewart J. Gapenne O. Di Paolo E. A. (Cambridge MA: MIT Press), 267.

[B4] Bottineau D. (2012). “Remembering voice past: Languaging as an embodied interactive cognitive technique,” in *Proceedings of the conference on interdisciplinarity in cognitive science research* (Moscow: RGGU [Russian State University for the Humanities]), 194–219.

[B5] Brewis J. (2014). The ethics of researching friends: On convenience sampling in qualitative management and organization studies. *Br. J. Manag.* 25 849–862. 10.1111/1467-8551.12064 25130355

[B6] Bruner J. S. (1985). *Child’s talk: Learning to use language.* New York, NY: W.W. Norton. 10.1177/026565908500100113

[B7] Buber M. (1970/1923). *I and Thou.* trans. Kaufmann W (New York, NY: Charles Scribner’s Sons).

[B8] Cameron C. (2022). *Climate activist dies after setting himself on fire at Supreme Court.* New York, NY: New York Times.

[B9] Candiotto L. De Jaegher H. (2021). Love in-between. *J. Ethics* 25 501–524. 10.1007/s10892-020-09357-9

[B10] Case A. Deaton A. (2020). *Deaths of despair and the future of capitalism.* Princeton, NJ: Princeton University Press. 10.1515/9780691199955

[B11] Colombetti G. (2011). Varieties of pre-reflective self-awareness: Foreground and background bodily feelings in emotion experience. *Inquiry* 54 293–313. 10.1080/0020174X.2011.575003

[B12] Colombetti G. (2014). *The feeling body: Affective science meets the enactive mind.* Cambridge, MA: MIT Press. 10.7551/mitpress/9780262019958.001.0001

[B13] Colombetti G. (2017). The embodied and situated nature of moods. *Philosophia (Ramat Gan)* 45 1437–1451. 10.1007/s11406-017-9817-0 30147178 PMC6086258

[B14] Cuffari E. C. (2020). On life-language continuity. *Constr. Found.* 15 149–151.

[B15] Cuffari E. C. Di Paolo E. De Jaegher H. (2015). From participatory sense-making to language: There and back again. *Phenomenol. Cogn. Sci.* 14 1089–1125.

[B16] Cummins F. (2019). *The ground from which we speak: Joint speech and the collective subject.* Newcastle upon Tyne: Cambridge Scholars Publishing.

[B17] Dahlberg K. Nyström M. Dahlberg H. (2007). *Reflective lifeworld research.* Lund: Studentlitteratur.

[B18] De Haan S. (2020). *Enactive psychiatry.* Cambridge: Cambridge University Press. 10.1017/9781108685214

[B19] De Jaegher H. Di Paolo E. (2007). Participatory sense-making. *Phenomenol. Cogn. Sci.* 6 485–507.

[B20] Di Paolo E. A. (2021). Enactive becoming. *Phenomenol. Cogn. Sci.* 20 783–809. 10.1007/s11097-019-09654-1

[B21] Di Paolo E. A. Cuffari E. C. De Jaegher H. (2018). *Linguistic bodies: The continuity between life and language.* Cambridge, MA: MIT press. 10.7551/mitpress/11244.001.0001

[B22] Elliott R. Timulak L. (2021). *Essentials of descriptive-interpretive qualitative research: A generic approach.* Washington, DC: American Psychological Association. 10.1037/0000224-000

[B23] Fitzgerald F. S. (1925). *The great gatsby.* New York, NY: Charles Scribner’s Sons

[B24] Fourlas G. N. (2022). *Anti-colonial solidarity: Race, reconciliation, and mena liberation.* Lanham, MD: Rowman & Littlefield Publishers.

[B25] Fuchs T. (2018). *Ecology of the brain: The phenomenology and biology of the embodied mind.* Oxford: Oxford University Press. 10.1093/med/9780199646883.001.0001

[B26] Gay R. (2019). *The case against hope.* New York, NY: The New York Times, 6.

[B27] Gravlee G. S. (2000). Aristotle on hope. *J. History Philos.* 38 461–477. 10.1353/hph.2005.0029 34409987

[B28] Harrison S. (2021). The feel of a recurrent gesture: Embedding the Vertical Palm within a gift-giving episode in China (aka the ‘seesaw battle’). *Gesture* 20 254–284.

[B29] Hodges B. H. (2007). Values define fields: The intentional dynamics of driving, carrying, leading, negotiating, and conversing. *Ecol. Psychol.* 19 153–178.

[B30] Hodges B. H. (2022). Values define agency: Ecological and enactive perspectives reconsidered. *Adaptive Behav.* 10.1177/10597123221076876

[B31] Hodges B. H. Baron R. M. (1992). Values as constraints on affordances: Perceiving and acting properly. *J. Theory Soc. Behav.* 22 263–294. 10.1111/j.1468-5914.1992.tb00220.x

[B32] Jemisin N. K. (2015). *The fifth season.* London: Orbit.

[B33] Jemisin N. K. (2016). *The obelisk gate.* London: Orbit.

[B34] Jemisin N. K. (2017). *The stone sky.* London: Orbit.

[B35] Kingsolver B. (2018). *Unsheltered.* London: Faber & Faber.

[B36] Kiverstein J. Miller M. Rietveld E. (2019). The feeling of grip: Novelty, error dynamics, and the predictive brain. *Synthese* 196 2847–2869. 10.1007/s11229-017-1583-9

[B37] Kravchenko A. (2011). How humberto maturana’s biology of cognition can revive the language sciences. *Constr. Found.* 6 352–362.

[B38] Kiverstein J. Rietveld E. (2021). Scaling-up skilled intentionality to linguistic thought. *Synthese* 198 175–194.

[B39] Krueger J. Maiese M. (2018). Mental institutions, habits of mind, and an extended approach to autism. *Thaumàzein Rivista Filosofia* 6 10–41.

[B40] Landgrebe J. (2012). I Think - You Know’ two epistemic stance markers and their significance in an innovation process. *Nordica Helsingiensia* 30 107–131.

[B41] Lear J. (2006). *Radical hope: Ethics in the face of cultural devastation.* Cambridge, MA: Harvard University Press. 10.4159/9780674040021

[B42] Lear J. (2021). Transience and hope: A return to Freud in a time of pandemic. *Int. J. Psychoanal.* 102 3–15. 10.1080/00207578.2021.1875836 33952008

[B43] Lecercle J. J. (2006). *A marxist philosophy of language.* Leiden: Brill. 10.1163/9789047408482

[B44] Lopez S. J. (2013). *Making hope happen: Create the future you want for yourself and others.* New York, NY: Simon and Schuster.

[B45] Lundquist C. (2008). Being torn: Toward a phenomenology of unwanted pregnancy. *Hypatia* 23 136–155. 10.1111/j.1527-2001.2008.tb01209.x

[B46] Machado de Oliveira V. (2021). *Hospicing Modernity: Facing humanity’s wrongs and the implications for social activism.* Berkeley, CA: North Atlantic Books.

[B47] Maiese M. (2022). Mindshaping, enactivism, and ideological oppression. *Topoi* 41 341–354. 10.1007/s11245-021-09770-1

[B48] Maiese M. Hanna R. (2019). *The mind-body politic.* Berlin: Springer. 10.1007/978-3-030-19546-5

[B49] Marcel G. (1973). *Tragic wisdom and beyond.* Evanston, IL: Northwestern University Press.

[B50] Martin A. (2013). *How we hope.* Princeton, NJ: Princeton University Press.

[B51] Martin A. (2019). “Interpersonal hope,” in *The moral psychology of hope*, eds Blöser C. Stahl T. (Lanham, MD: Rowman and Littlefield), 229–248.

[B52] Maturana H. R. (1978). “Biology of language: The epistemology of reality,” in *Psychology and biology of language and thought. Essays in honor of eric lenneberg*, eds Miller G. Lenneberg E. (New York, NY: Academic Press), 27–63.

[B53] Maturana H. R. (1983). What is it to see? *Arch. Biol. Med. Exp.* 16 255–269. 6680992

[B54] McGreer V. (2004). The art of good hope. *Ann. Am. Acad. Polit. Soc. Sci.* 592 100–127. 10.1177/0002716203261781

[B55] McNally D. (2001). *Bodies of meaning: Studies on language, labor, and liberation.* Albany, NY: SUNY Press.

[B56] Michener R. T. (2020). “Post-kantian to postmodern considerations of (Theological) hope,” in *Historical and multidisciplinary perspectives on hope*, ed. van den Heuvel S. C. (Cham: Springer), 77. 10.1007/978-3-030-46489-9_5

[B57] Morton T. (2018). *Being ecological.* Cambridge, MA: MIT Press. 10.7551/mitpress/11638.001.0001

[B58] Overall C. (2016). “Paradox in Practice,” in *New philosophies of sex and love: Thinking through desire*, eds Adams S. L. Davidson C. M. Lundquist C. R. (Lanham, MD: Rowman & Littlefield), 145.

[B59] Pettit P. (2004). Hope and its place in mind. *Ann. Am. Acad. Polit. Soc. Sci.* 592 152–165. 10.1177/0002716203261798

[B60] Quintero A. M. De Jaegher H. (2020). Pregnant agencies: Movement and participation in maternal–fetal interactions. *Front. Psychol.* 11:1977. 10.3389/fpsyg.2020.01977 32922337 PMC7456916

[B61] Raimondi V. (2014). Social interaction, languaging and the operational conditions for the emergence of observing. *Front. Psychol.* 5:899. 10.3389/fpsyg.2014.00899 25177308 PMC4132289

[B62] Raimondi V. (2019). The bio-logic of languaging and its epistemological background. *Lang. Sci.* 71 19–26.

[B63] Raimondi V. (2021). The operational matrix of languaging: A radically relational understanding of language. *Riv. Ital. Filos. Ling.* 15 49–58.

[B64] Rankine C. (2014). *Citizen: An American lyric.* Minneapolis, MN: Graywolf Press.

[B65] Reed E. S. (1996). *Encountering the world: Toward an ecological psychology.* Oxford: Oxford University Press. 10.1016/S0166-4115(05)80023-8

[B100] Rorty R. (2000). “Universality and truth,” in *Rorty and his critics, (Ser. Philosophers and their critics)*, ed. R. Brandom (Oxford: Blackwell), 9.

[B66] Snyder C. R. (2002). Hope theory: Rainbows in the mind. *Psychol. Inq.* 13 249–275. 10.1207/S15327965PLI1304_01

[B67] Solnit R. (2016). *Hope in the dark: Untold histories, wild possibilities.* Chicago, IL: Haymarket Books.

[B68] Solnit R. (2020). *The impossible has already happened: What coronavirus can teach us about hope.* London: The Guardian, 7.

[B69] Stawarska B. (2009). *Between you and i: Dialogical phenomenology.* Athens, OH: Ohio University Press. 10.1353/book.6990

[B70] Stewart J. (2010). “Foundational issues in enaction as a paradigm for cognitive science: From the origin of life to consciousness and writing,” in *Enaction: Toward a new paradigm for cognitive science*, eds Stewart J. Gapenne O. Di Paolo E. A. (Cambridge MA: MIT Press), 1–31.

[B71] Stitzlein S. (2019). “Pragmatist hope,” in *The moral psychology of hope*, eds Blöser C. Stahl T. (London: Rowman & Littlefield), 93–112.

[B72] Thibault P. J. (2011). First-order languaging dynamics and second-order language: The distributed language view. *Ecol. Psychol.* 23 210–245.

[B73] Thompson E. (2021). *Enacting hope.* Santa Fe, NM: Upaya Institute and Zen Center Dharma.

[B74] Tillmann-Healy L. M. (2003). Friendship as method. *Qual. Inq.* 9 729–749. 10.1177/1077800403254894

[B75] van den Herik J. (2019). *Talking about talking: An ecological-enactive perspective on language.* Rotterdam: Erasmus University Rotterdam.

[B76] van den Herik J. C. (2021). Rules as resources: An ecological-enactive perspective on linguistic normativity. *Phenomenol. Cogn. Sci.* 20 93–116.

[B77] Van den Heuvel S. C. (2020). *Historical and multidisciplinary perspectives on hope.* Berlin: Springer Nature. 10.1007/978-3-030-46489-9

[B78] Varela F. J. (1999). *Ethical know-how: Action, wisdom, and cognition.* Redwood City, CA: Stanford University Press.

[B79] Welch S. (2022). *Choreography as embodied critical inquiry: Embodied cognition and creative movement.* Berlin: Springer Nature.

[B80] West C. (2004). “Prisoners of hope,” in *The impossible will take a little while*, ed. Loeb P. R. (Cambridge, MA: Basic Books), 296.

[B101] West C. (2008). *Hope on a tightrope.* Carlsbad, CA: Hay House, Inc.

[B81] Woolfrey J. (2016). The primacy of hope. *Soc. Philos. Today* 32 125–136. 10.5840/socphiltoday2016101335

[B82] Zhong R. (2022). *These climate scientists are fed up and ready to go on strike.* New York, NY: The New York Times.

